# Targeted Induction of Endoplasmic Reticulum Stress Induces Cartilage Pathology

**DOI:** 10.1371/journal.pgen.1000691

**Published:** 2009-10-16

**Authors:** M. Helen Rajpar, Ben McDermott, Louise Kung, Rachel Eardley, Lynette Knowles, Mel Heeran, David J. Thornton, Richard Wilson, John F. Bateman, Richard Poulsom, Peter Arvan, Karl E. Kadler, Michael D. Briggs, Raymond P. Boot-Handford

**Affiliations:** 1 Wellcome Trust Centre for Cell-Matrix Research, Faculty of Life Sciences, The University of Manchester, Manchester, United Kingdom; 2 Musculoskeletal Disorders Theme, Murdoch Children's Research Institute, Royal Children's Hospital, Parkville, Victoria, Australia; 3 Histopathology Unit, Cancer Research UK, London Research Institute, London, United Kingdom; 4 Division of Metabolism, Endocrinology, and Diabetes, University of Michigan Medical School, Ann Arbor, Michigan, United States of America; Children's Hospital Boston and Harvard Medical School, Howard Hughes Medical Institute, United States of America

## Abstract

Pathologies caused by mutations in extracellular matrix proteins are generally considered to result from the synthesis of extracellular matrices that are defective. Mutations in type X collagen cause metaphyseal chondrodysplasia type Schmid (MCDS), a disorder characterised by dwarfism and an expanded growth plate hypertrophic zone. We generated a knock-in mouse model of an MCDS–causing mutation (*COL10A1* p.Asn617Lys) to investigate pathogenic mechanisms linking genotype and phenotype. Mice expressing the collagen X mutation had shortened limbs and an expanded hypertrophic zone. Chondrocytes in the hypertrophic zone exhibited endoplasmic reticulum (ER) stress and a robust unfolded protein response (UPR) due to intracellular retention of mutant protein. Hypertrophic chondrocyte differentiation and osteoclast recruitment were significantly reduced indicating that the hypertrophic zone was expanded due to a decreased rate of VEGF–mediated vascular invasion of the growth plate. To test directly the role of ER stress and UPR in generating the MCDS phenotype, we produced transgenic mouse lines that used the collagen X promoter to drive expression of an ER stress–inducing protein (the cog mutant of thyroglobulin) in hypertrophic chondrocytes. The hypertrophic chondrocytes in this mouse exhibited ER stress with a characteristic UPR response. In addition, the hypertrophic zone was expanded, gene expression patterns were disrupted, osteoclast recruitment to the vascular invasion front was reduced, and long bone growth decreased. Our data demonstrate that triggering ER stress *per se* in hypertrophic chondrocytes is sufficient to induce the essential features of the cartilage pathology associated with MCDS and confirm that ER stress is a central pathogenic factor in the disease mechanism. These findings support the contention that ER stress may play a direct role in the pathogenesis of many connective tissue disorders associated with the expression of mutant extracellular matrix proteins.

## Author Summary

Mutations in genes for extracellular matrix proteins are generally thought to exert their pathogenic effects because of resulting defects in extracellular matrix. However, it is becoming increasingly clear that such mutations can also have significant effects inside the cell due to the induction of ER stress. Mutations in type X collagen cause a dwarfism called metaphyseal chondrodysplasia type Schmid. A gene targeted mouse model expressing mutant type X collagen exhibited an expanded hypertrophic zone of the growth plate and significant increases in cellular ER stress, as noted previously. VEGF expression was disrupted leading to decreases in the rate of vascular invasion. To directly assess the role of elevated ER stress in disease pathogenesis, transgenic mouse lines expressing an exogenous, ER stress–inducing protein (*cog* mutant of thyroglobulin—Tg^cog^) targeted to hypertrophic chondrocytes were generated. Mice expressing Tg^cog^ protein showed elevated ER stress, an expanded hypertrophic zone, and reduced bone growth demonstrating that elevated ER stress and the resultant UPR is the principal pathogenic mechanism causing this cartilage pathology. It is possible that therapeutic strategies aimed at alleviating ER stress may be beneficial in this and other connective tissue diseases caused by mutant extracellular matrix genes.

## Introduction

Most bones in the body grow by a process of endochondral ossification in which a cartilage template is laid down and subsequently converted to bone. The process takes place in well-defined growth plates at the ends of long bones. Longitudinal bone growth is achieved in a tightly controlled process in which chondrocytes first proliferate forming columns, then exit the cell cycle, hypertrophy and mineralise [1],[2]. Collagen X is expressed solely and specifically by hypertrophic chondrocytes although the precise function of this protein in bone growth remains controversial. Roles for collagen X as an extracellular scaffold or in the mineralisation of hypertrophic growth plate have been proposed and yet most studies have shown that gene ablation does not produce an overt phenotype in mice [3],[4]. Terminally differentiated hypertrophic chondrocytes die by programmed cell death as blood vessels invade the calcified region of the growth plate. The vasculature introduces osteoclasts which erode the mineralised cartilage matrix and osteoblasts which replace the cartilage matrix with bone. The differentiation process is controlled by a cascade of growth factors including PTHrP, Indian Hedgehog and VEGF [2],[5],[6].

Mutations in cartilage extracellular matrix (ECM) proteins such as collagens II, IX, X and XI, aggrecan, COMP and matrilin 3 disrupt growth plate differentiation and cause chondrodysplasia [7]–[9]. In these types of dysplasia, it is generally thought that the pathology arises as a result of an ECM that is either defective due to the incorporation of the mutant protein or alternatively, deficient in the protein due to intracellular degradation of the mutated protein prior to secretion. However, it is becoming increasingly clear that the synthesis of mutant proteins destined for the ECM can trigger ER stress in affected cells eliciting an UPR [10]–[18]. The UPR is orchestrated through the interaction of the molecular chaperone BiP (Grp78) with a set of transmembrane ER stress sensors (IRE1, ATF6, PERK). BiP normally binds to these membrane bound sensors but as ER stress increases, BiP is sequestered into the ER lumen by interaction with accumulating unfolded proteins. Loss of BiP binding causes the stress sensors to activate resulting in downstream events such as Xbp-1 splicing, ATF6 cleavage, eIF2α phosphorylation and ATF4 translation (extensively reviewed in [19]–[22]). As a result, the synthesis of stress related proteins such as chaperones like BiP are increased to enhance the protein folding capacity of the ER. General protein synthesis is decreased through phospho eIF2α-dependant translational suppression of 5′ cap containing mRNAs to slow down the entry of unfolded protein into the ER. In addition, relevant pathways for disposing of misfolded proteins such as ER associated degradation or autophagy are up-regulated.

Mutations in type X collagen, predominantly clustered in the C-terminal non collagenous (NC1) domain that is required for collagen trimer assembly, cause MCDS, a relatively mild form of dwarfism [4],[23],[24]. Two recent studies of transgenic mouse lines expressing MCDS-associated mutations, a 13 bp deletion in the NC1 coding region of the gene [11] and the mouse equivalent of the *COL10A1* p.Pro620fsX621 mutation [13], have reported a UPR in the collagen X-expressing hypertrophic zone of the growth plate and provided strong evidence for ER stress playing an important role in the pathogenesis of this disease. We generated a knock-in mouse model of the *COL10A1* p.Asn617Lys MCDS-causing mutation and demonstrate that the MCDS-associated expanded hypertrophic zone [11],[13] occurred because of disrupted VEGF-mediated osteoclast erosion of the mineralised cartilage at the vascular invasion front. ER stress and a strong UPR provoked by misfolding mutant collagen X were key features of the hypertrophic chondrocytes in the MCDS mouse. Furthermore the targeted stimulation of ER stress in hypertrophic chondrocytes *in vivo* was sufficient to replicate the MCDS growth plate phenotype functionally demonstrating the central role played by ER stress in the disease mechanism.

## Results

### Generation of a collagen X (p.Asn617Lys) knock-in mouse model of MCDS

**Figure 1:**
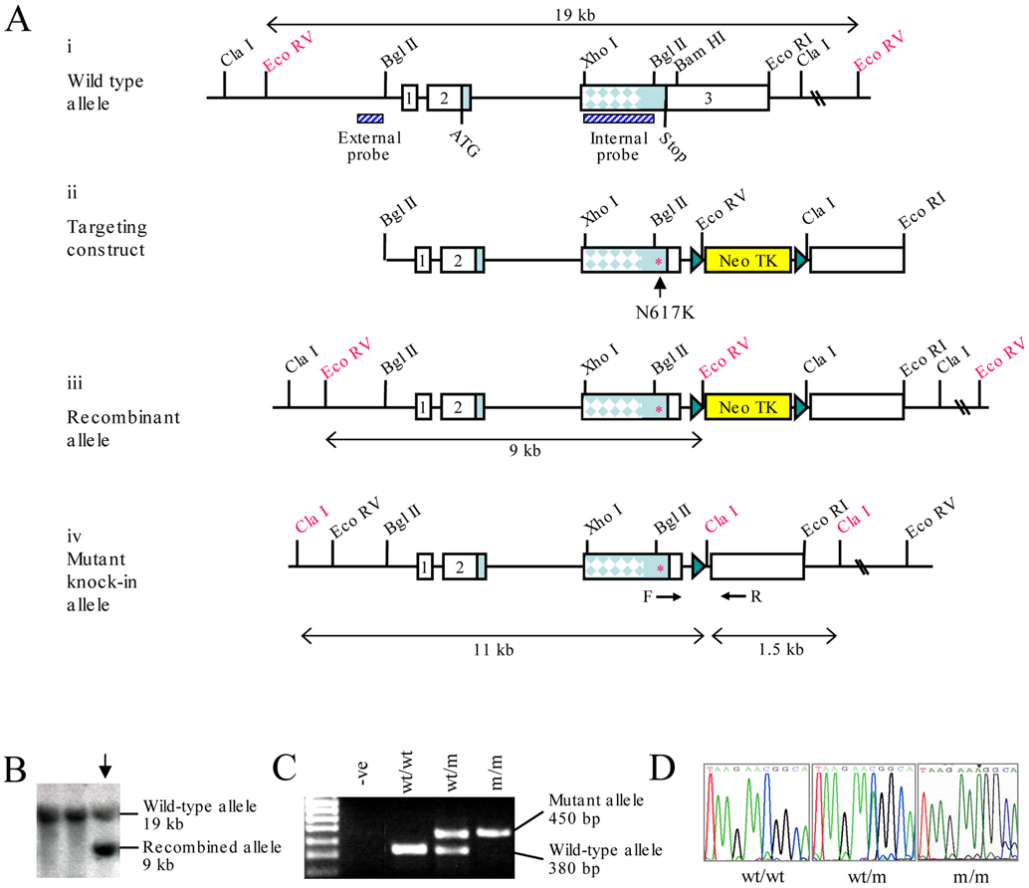
Generation of the *Col10a1* p.Asn617Lys MCDS mouse model. (A) Targeting strategy: (i) Wildtype collagen X allele indicating location of probes used for Southern blotting and relevant restriction enzyme sites; exons are numbered boxes and the region of exon 3 encoding the collagenous domain is indicated by dappling; (ii) Targeting construct with p.Asn617Lys (N617K) mutation (*) and floxed Neo TK selection cassette; (iii) Recombinant allele identified using the external probe; (iv) Mutant knock-in allele containing the p.Asn617Lys mutation and residual *LoxP* site following deletion of the Neo TK cassette by transient *Cre* transfection and FIAU selection. (B) Positive clones were selected by screening EcoRV digested DNA with the external probe. Southern blot analysis was used to identify homologously recombined clones (↓) containing both the wildtype allele (19 kb) and the recombinant allele (9 kb). (C) Genotyping was performed by PCR amplification across the residual *LoxP* site using F and R primers shown in (A). (D) Chromatograph of the sequence flanking the mutation site from F2 mice, showing the C>A single base pair substitution in the heterozygote (wt/m), represented by a double peak, and the single A peak in the mouse homozygous for the mutation (m/m).

Mice harbouring the p.Asn617Lys mutation in the NC1 domain of collagen X that causes MCDS in humans were generated following homologous recombination in R1 ES cells. The AAC→AAA codon change was introduced into exon 3 of the *Col10a1* gene by site directed mutagenesis. Of the 360 G418 resistant ES cell clones selected and analysed, 7 were shown to be homologously recombined by Southern blotting (Figure 1B) and 5 of these were found to contain the mutation by direct sequencing (data not shown). Three correctly targeted clones were transiently transfected with an expression vector containing *cre* recombinase to delete the floxed selection cassette. Germline chimeras were generated from one of these clones. The mutant allele was detected by a PCR assay (Figure 1C) and the presence of the mutated base causing the p.Asn617Lys substitution confirmed by direct sequencing of the genomic DNA of mutant offspring (Figure 1D).

Mice heterozygous and homozygous for the substitution will now be referred to as wt/m and m/m respectively, with wildtype littermates being denoted as wt/wt.

### Knock-in mice develop a short-limbed dwarfism

**Figure 2:**
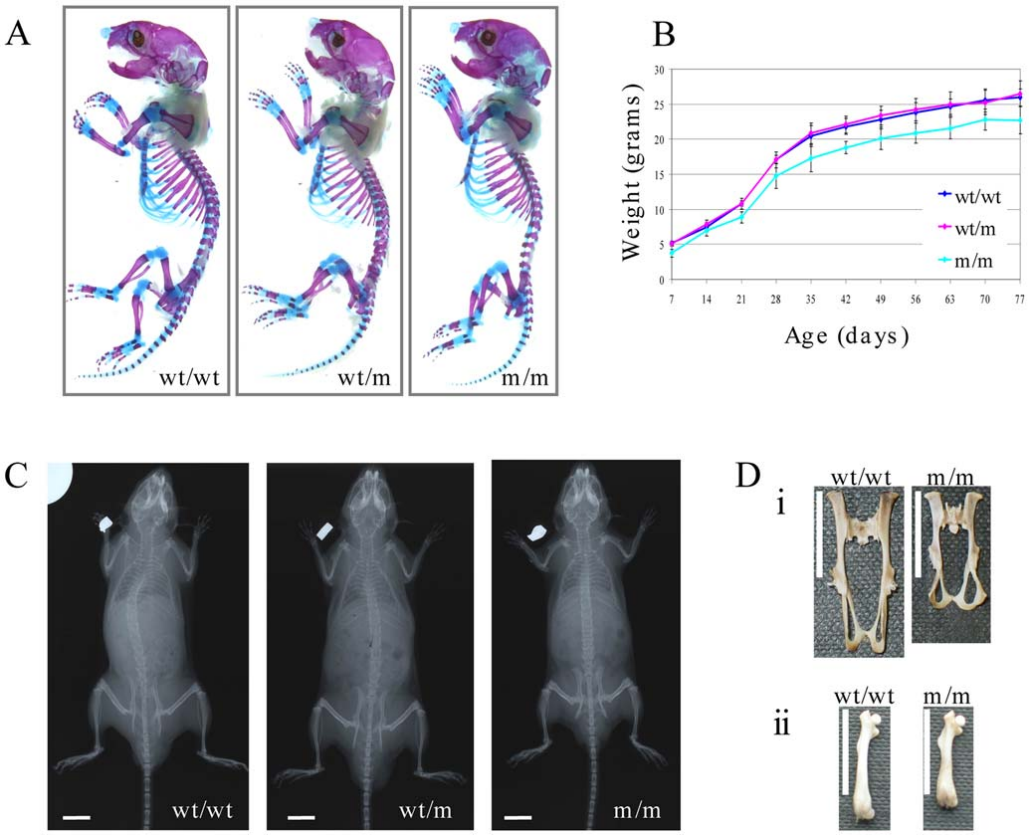
Macroscopic analyses of the MCDS mouse phenotype. (A) Skeletal preps of new born mice stained with alizarin red (bone) and alcian blue (cartilage). (B) Growth curves of mice (females) heterozygous (wt/m) and homozygous (m/m) for collagen X p.Asn617Lys mutation, compared to wild-type (wt/wt) litter mates. (C) X-ray images of male mice at 11 weeks of age, scale bar = 1 cm. (D) Comparison of bone lengths between wt/wt and m/m mice: (i) pelvis; (ii) femur, vertical white scale bar = 1 cm.

Neither wt/m or m/m mice had any overt skeletal phenotype at birth (Figure 2A). The growth rates of wt/m and wt/wt mice were indistinguishable whereas the m/m mice were growth retarded (Figure 2B; Table 1).

**Table 1:** Body measurements of *Col10a1* p.Asn617Lys mutant mice.

Measurement	Genotype	New born	1 week	2 week	3 week	10 week
Body weights (g)	wt/wt	-	5.2±0.3 (4)	6.9±0.7 (4)	10.8±0.4 (4)	25.5±1.6 (7)
	wt/m	-	5.1±0.3 (6)	7.9±0.5 (6)	10.8±0.8 (6)	25.3±1.7 (14)
	m/m	-	3.7±0.5 (7) **	4.8±0.7 (7) *	8.8±0.8 (7) **	22.8±1.5 (8) **
Femur Length (mm)	wt/wt	2.1±0.1 (4)	5.0±0.1 (7)	7.7±0.1 (4)	8.2±1.5 (7)	13.7±0.4 (4)
	wt/m	2.1±0.1 (5)	4.9±0.1 (11)	7.1±0.1 (6) ^†^	7.5±1.4 (7) ^†^	12.7±0.5 (6)
	m/m	2.0±0.1 (6)	4.3±0.1 (7) ^†† #^	6.1±0.1 (4) **	6.9±1.2 (7) ^†† #^	11.0±0.3 (4) *
Pelvis Length (mm)	wt/wt	3.2±0.1 (4)	8.3±0.2 (7)	10.6±0.5 (4)	10.6±0.2 (7)	17.9±0.4 (4)
	wt/m	3.3±0.1 (5)	7.8±0.1 (11)	10.2±0.1 (6)	9.8±0.2 (7)	17.7±0.2 (6)
	m/m	2.9±0.1 (6)	6.6±0.2 (7) *	8.2±0.1 (4) **	8.8±0.2 (7) ^†† #^	15.5±0.1 (4) **

Mean±SEM (n): *p<0.05 when compared to wt and wt/m; **p<0.001 when compared to wt/wt and wt/m; ^†^p<0.05 when compared to wt/wt; ^††^p<0.001 when compared to wt/wt; ^#^p<0.05 when compared to wt/m.

Skeletal analyses at 11 week of age revealed that m/m mice had shorter endochondral bones than their wt/wt littermates (Figure 2C and 2D). The femurs of m/m animals were between 14 and 21% shorter than wt/wt controls over the time course of the experiment (Table 1). The lengths of femurs in the wt/m mice were intermediate between the wt/wt and m/m values from 2 weeks of age onwards and the reduction in bone lengths in wt/m animals reached statistical significance at 2 and 3 weeks of age when compared to control wt/wt mice (p<0.05; Table 1). The differences between wt/wt and m/m femur length were maintained at 1 year of age (mean±SEM (n): wt/wt 16.3±0.3 mm (5); wt/m 15.6±0.7 mm (6); m/m 13.6±1.3 mm (7); wt/wt or wt/m versus m/m p<0.01). Pelvis shape was distorted (Figure 2C and 2D) and pelvis length significantly shorter in m/m compared to wt/wt mice from 1 week of age. The pelvic distortion was not apparent in wt/m mice but there was a detectable, albeit not significant, decrease in pelvis length compared to wt/wt mice at most ages examined (Figure 2C; Table 1). The skull, which is formed largely by intramembranous- rather than endochondral- ossification was unaffected by genotype (Figure 2C) with inner canthal distances at 10 weeks of age being wt/wt 0.77±0.01 mm, wt/m 0.81±0.01 mm and m/m 0.81±0.03 mm (no significant differences).

### Growth plate hypertrophic zone expansion in mice expressing mutant collagen X

**Figure 3:**
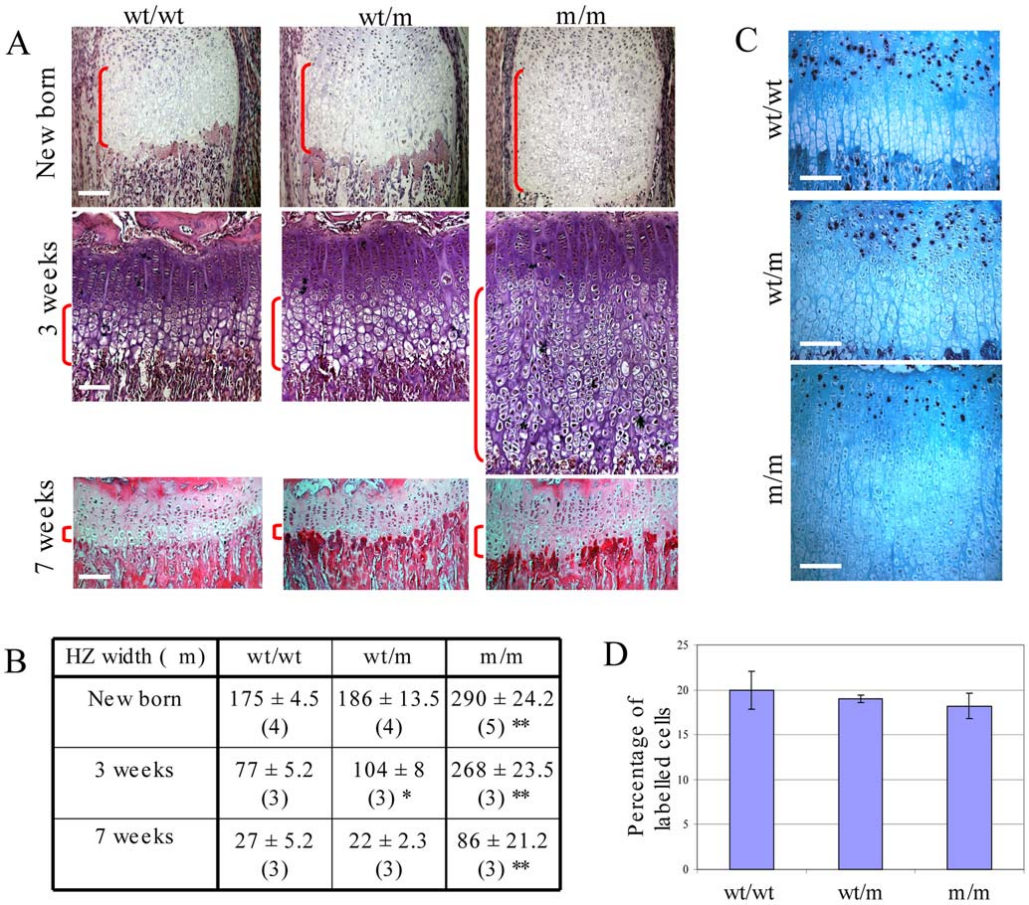
Analysis of hypertrophic zone width. (A) H & E staining of tibial growth plates from new born, 3 week and 7 week old MCDS and control mice. The hypertrophic zone is indicated by the vertical red brackets; horizontal white scale bar = 100 µm. (B) Hypertrophic zone widths at specified time points. *p<0.05, **p<0.01 versus control (wt/wt). (C) 5-bromo-2′-deoxyuridine (BrdU) labelling of proliferative cells in the growth plate of 3 week old mice. Positive cells stained black. (D) Graph showing the percentage of BrdU positive cells within the proliferative zone. The number of labelled cells in 4 different sections from 5 wt/wt, 5 wt/m and 4 m/m animals were counted.

The hypertrophic zones of tibial growth plates were markedly expanded in m/m animals (Figure 3A). Such expansions were also apparent in growth plates from other bones such as the femur and ribs (data not shown). The expansion of the hypertrophic zone was apparent at birth in m/m compared to wt/wt animals and was still apparent at 7 weeks of age even though the overall width of the growth plate in all genotypes decreased by approximately 70% over this period (Figure 3A and 3B). In wt/m mice, a significant (approx. 35%) expansion of the hypertrophic zone was apparent at 3 weeks of age but had resolved by 7 weeks of age (Figure 3A and 3B). There was no overt effect upon the pattern of trabecular bone deposition beneath the expanded growth plates in mutant mice (Figure 3A; 7 week samples).

No significant differences in the chondrocyte proliferation rates were detected between genotypes (Figure 3C and 3D) ruling out the possibility that increased proliferation of chondrocytes contributed to the expanded hypertrophic zone in mice expressing mutant collagen X.

### Collagen X secretion is delayed and hypertrophic chondrocyte differentiation disrupted in mutant mice

**Figure 4:**
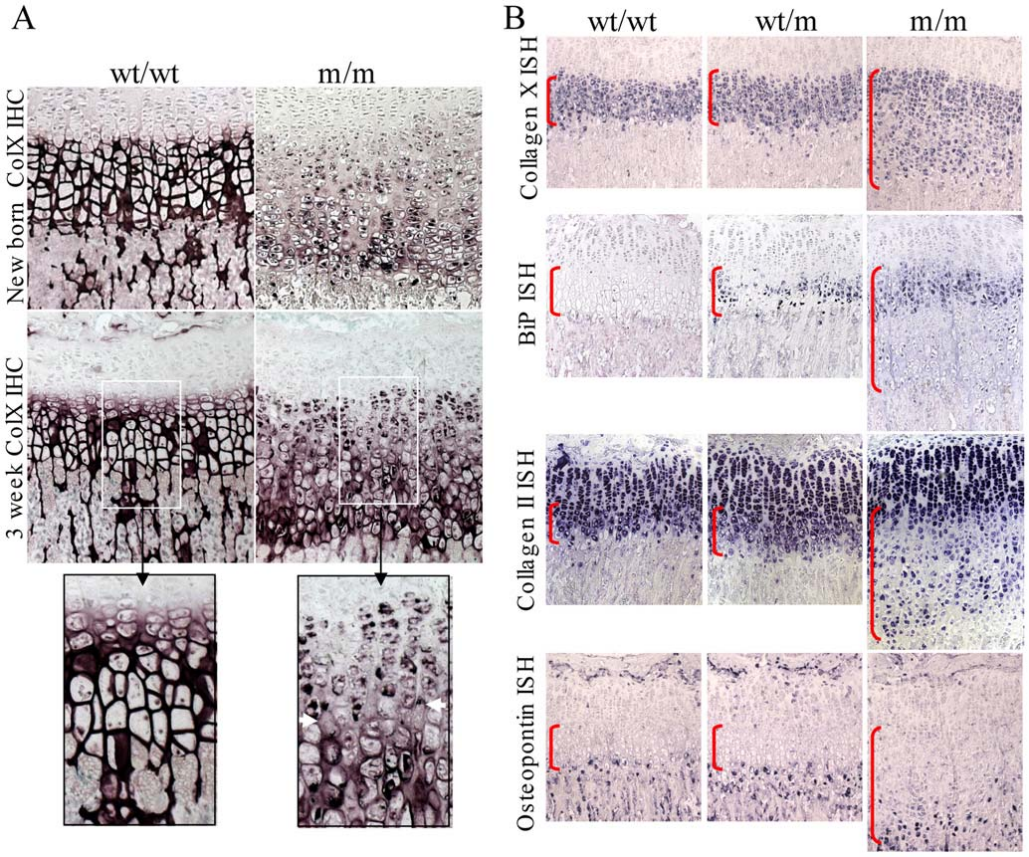
Histological characterisation of 3-week-old tibial growth plates. (A) Collagen X immunohistochemistry for MCDS and control mice. Purple staining indicates collagen X localisation; The black boxed photomicrographs represent an expanded view of the indicated areas in the sections from 3 week old mice. The intracellular accumulation of collagen X in the m/m growth plates is apparent in the upper hypertrophic zone. The transition region in which intracellular accumulation of collagen X resolves accompanied by a limited secretion of mutant collagen X is indicated by the white horizontal arrows in the expanded view of the 3 week m/m sample. (B) In situ hybridisation for *Col10a1*, *BiP*, *Col2a1* (collagen II) and *Opn* (osteopontin) in tibial growth plate sections from 3 week old animals. The presence of the transcript is indicated by blue staining. The hypertrophic zone is indicated by the vertical red brackets.

Immunolocalisation of collagen X revealed the expected extracellular staining in the hypertrophic zones of control growth plates with no obvious intracellular staining (Figure 4A; wt/wt). However, the secretion of mutant collagen X was reduced and significantly delayed in m/m mice with marked intracellular retention of the mutant protein apparent in the upper hypertrophic zone (Figure 4A). Immunolocalisation of collagen X in wt/m animals appeared unaffected compared to wt/wt apart from an occasional hypertrophic cell showing some intracellular immunoreactivity (data not shown).

*In situ* analysis revealed that collagen X mRNA expression was increased in hypertrophic chondrocytes of wt/wt mice (Figure 4B). Wt/m mice exhibited an expression pattern similar to that of the wt/wt controls. m/m mice also showed collagen X induction in the upper hypertrophic zone but this was followed by down-regulation and then re-expression producing a ‘speckled’ appearance in the lower hypertrophic zone (Figure 4B). The expression of BiP mRNA was below the level of detection in wt/wt controls whereas it is clearly up-regulated in the hypertrophic zone of wt/m mice. In m/m mice, BiP expression was markedly up-regulated in the upper hypertrophic zone and expression became sporadic in the lower hypertrophic zone similar to that seen for collagen X expression in the mutant mice. In wt/wt and wt/m mice, collagen II expression was down-regulated in the hypertrophic compared to proliferative zone. In m/m mice, collagen II mRNA was down-regulated in the upper hypertrophic zone but became strongly up-regulated in scattered cells in the lower portion of elongated hypertrophic zone (Figure 4B). Expression of osteopontin, a marker of terminal hypertrophic chondrocyte differentiation, was restricted to a narrow region of hypertrophic chondrocytes at the base of the growth plate in wt/wt and wt/m mice but was expressed by individual cells scattered throughout the lower half of the elongated hypertrophic zone in m/m mice (Figure 4B).

### Chondrocytes expressing the mutant collagen X exhibit increased ER stress and the UPR

**Figure 5:**
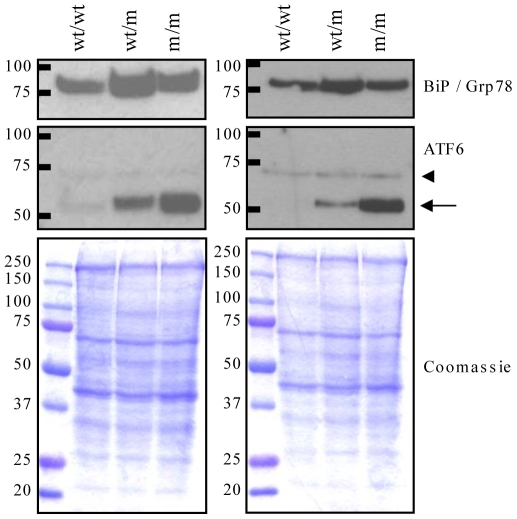
Western blot analysis of MCDS mouse growth plate extracts. The panels represent two independent analyses of growth plate extracts from wildtype (wt/wt), heterozygote (wt/m) and mice homozygous (m/m) for the *Col10a1* p.Asn617Lys mutation. For each analysis, growth plates from the ribs of two 21 day old mice were pooled and extracted in sample buffer as described in the methods. Twenty micrograms of each extract was resolved on SDS-PAGE gels under reducing conditions, and western blotted for BiP and ATF6. Coomassie blue stained gels are protein loading controls. For the ATF6 blot, the cleaved form (50 kDa indicated by arrow) was readily detected in wt/m and m/m mouse extracts whereas the full-length ATF6 (90 kDa) was not detected in any samples. A non-specific band at about 70 kDa (arrow head) was apparent.

The intracellular retention of mutant collagen X (Figure 4A) and the up-regulation of BiP mRNA (Figure 4B) are signs that the hypertrophic chondrocytes are experiencing increased levels of ER stress. We therefore conducted western blotting analyses of rib growth plate extracts to biochemically characterize this cellular response. The levels of BiP protein were higher in both wt/m and m/m extracts than those from wt/wt growth plate (Figure 5). Full length ATF6 (∼90 kDa) was not detectable in control extracts (Figure 5; wt/wt). However, the cleaved ∼50 kDa active form of ATF6 was readily detected in both wt/m and m/m growth plate extracts inferring that ATF6 was significantly induced and activated in hypertrophic chondrocytes expressing the mutant collagen X.

### Hypertrophic zone expansion occurs because of disrupted VEGF expression and a resulting decreased recruitment of osteoclasts to the vascular invasion front

**Figure 6:**
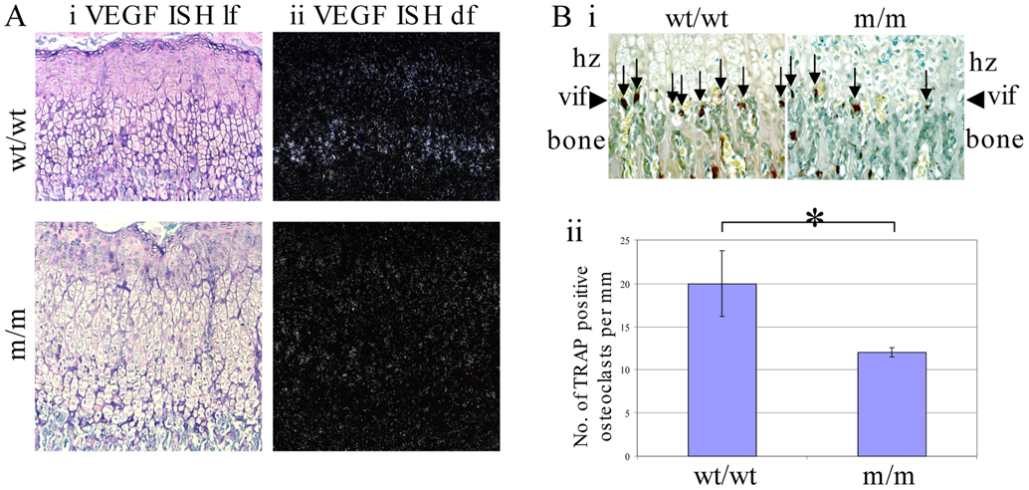
VEGF expression and osteoclast recruitment at the vascular invasion front. (A) VEGF *in situ* hybridisation by ^35^S-labelled riboprobe and autoradiography: (i) light-field and (ii) dark field view of the same regions of the growth plate sections. (B) TRAP staining for osteoclasts at the vascular invasion front (vif - arrow heads, hz = hypertrophic zone); (i) Vertical arrows indicate positively stained cells (red/brown colour); (ii) Histogram depicting number of positive cells per 1 mm of vascular invasion front. *p<0.01 (n = 3 for each genotype).

VEGF expression by hypertrophic chondrocytes has previously been shown to play an important role in mediating the width of the hypertrophic zone through modulating the rate of vascular invasion [6],[25]. VEGF expression was readily detected in wildtype growth plate where expression was localised to the lower half of the hypertrophic zone. No such synchronised expression was detected in the expanded hypertrophic zone of the m/m mice (Figure 6A). It therefore appears that VEGF can be added to the list of genes described above (Figure 4) and previously [11],[13] whose expression by hypertrophic chondrocytes is disrupted as a result of mutant collagen X expression. Vascular invasion of the base of the growth plate introduces osteoclasts to the vicinity and it is these cells that are responsible for eroding the cartilage matrix allowing its replacement by bone. Tartrate-resistant acid phosphatase (TRAP) staining for osteoclasts revealed significantly fewer osteoclasts localised to the vascular invasion front in m/m compared to wt/wt growth plates (p<0.01; Figure 6B).

### Targeted expression of the Tg^cog^ protein in hypertrophic chondrocytes induces increased ER stress and the UPR

The expression of MCDS-causing collagen X mutations *in vivo* results in an expanded hypertrophic zone with cells exhibiting a robust UPR which has been proposed to play a key role in disease pathogenesis ([11],[13] and data presented above). In order to directly test the role of UPR in the disease mechanism, we generated a mouse in which ER stress would be targeted to hypertrophic chondrocytes independently from the expression of a mutant collagen X protein.

**Figure 7:**
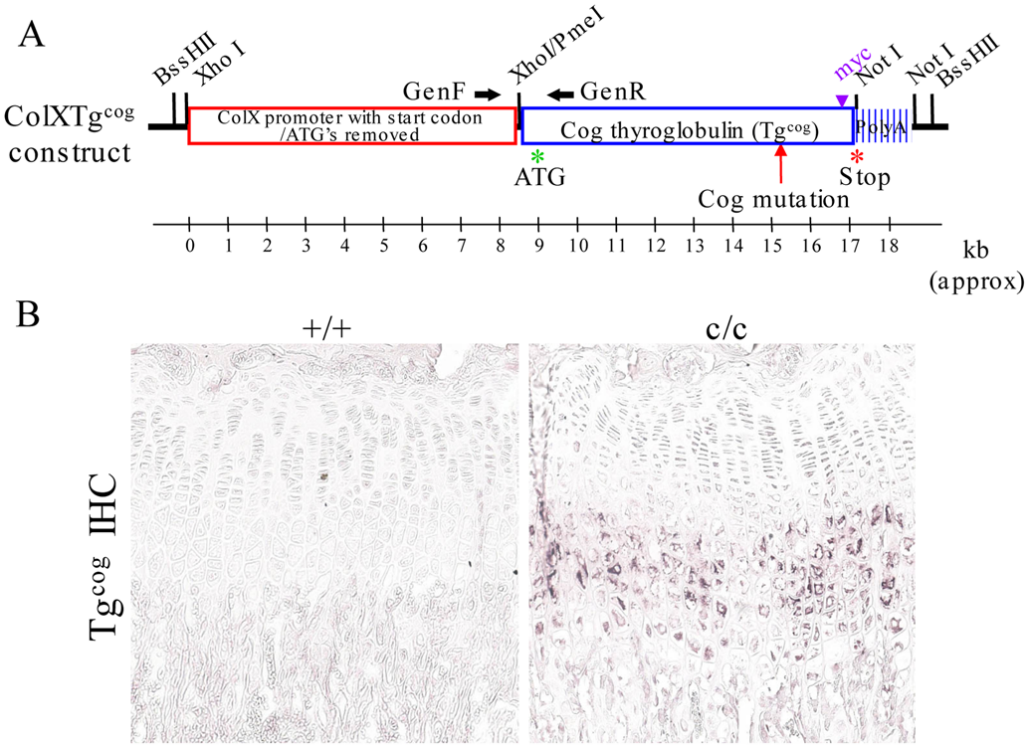
Generation of the *ColXTg^cog^* mouse. (A) Schematic of the *ColXTg^cog^* construct. The collagen X promoter as ligated upstream of the cDNA sequence encoding the myc-tagged *cog* mutant form of thyroglobulin (Tg^cog^). The cloning vector was removed and the construct injected into mouse embryo pronuclei; (B) Immunohistochemical localisation of Tg^cog^ using a myc antibody on tibial growth plate section from a 3 week old control (+/+) and a transgenic *ColXTg^cog^* littermate (c/c). Expression of Tg^cog^ is limited to the hypertrophic zone of the transgenic (c/c) mouse.

Thyroglobulin is the precursor protein for the generation of thyroid hormones and its expression is restricted to thyroid cells. The *cog* mutant of thyroglobulin (Tg^cog^) provokes a strong UPR due to its inability to dimerise in the ER, an obligate step for thyroglobulin secretion. As a result, Tg^cog^ is efficiently retained intracellularly [26],[27]. The expression of Tg^cog^ was targeted to hypertrophic chondrocytes through coupling to the collagen X promoter (*ColXTg^cog^*, Figure 7A). Transgenic mice were created by pronuclear injection and 2 independent lines that expressed the Tg^cog^ protein in hypertrophic chondrocytes (Figure 7B) were investigated further and gave similar results. One mouse line had approximately 5 copies of the transgene at a single insertion site and expressed 17% and 33% the level of endogenous collagen X in the hemizygous and homozygous state, respectively (results of collagen X and transgene qPCR not shown). This line was used to generate the data presented herein. Hemizygous and homozygous transgenic mice will be referred to as +/c and c/c respectively, and wildtype littermate controls as +/+.

**Figure 8:**
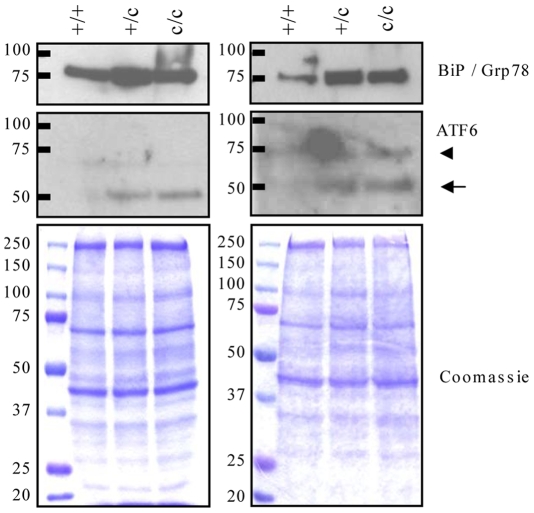
Western blot analysis of *ColXTg^cog^* mouse growth plate extracts. The panels represent two independent analyses of growth plate extracts from wildtype (+/+), hemizygous (+/c) and mice homozygous (c/c) for *ColXTg^cog^* transgene. For each analysis, growth plates from the ribs of two 21 day old mice were pooled and extracted in sample buffer as described in the methods. Twenty micrograms of each extract was resolved on SDS-PAGE gels under reducing conditions, and western blotted for BiP and ATF6. Coomassie blue stained gels are protein loading controls. For the ATF6 blot, the cleaved form (50 kDa indicated by arrow) was readily detected in +/c and c/c mouse extracts whereas the full-length ATF6 (90 kDa) was not detected in any samples. A non-specific band at about 70 kDa (arrow head) was apparent.

BiP protein was elevated in +/c or c/c rib growth plate extracts when compared with +/+ controls (Figure 8). As noted previously (Figure 5), full-length (∼90 kDa) ATF6 was not detectable in wildtype controls but the cleaved activated form of the protein (∼50 kDa) was markedly induced in both +/c and c/c mice (Figure 8). The increased levels of BiP and cleaved ATF6 are clear indications that the expression of Tg^cog^ in hypertrophic chondrocytes elevated ER stress and induced an UPR with similar characteristics to that caused by the mutant collagen X (cf Figure 5 and Figure 8).

### The expression of Tg^cog^ protein in hypertrophic chondrocytes causes an expanded hypertrophic zone and decreased bone growth

**Figure 9:**
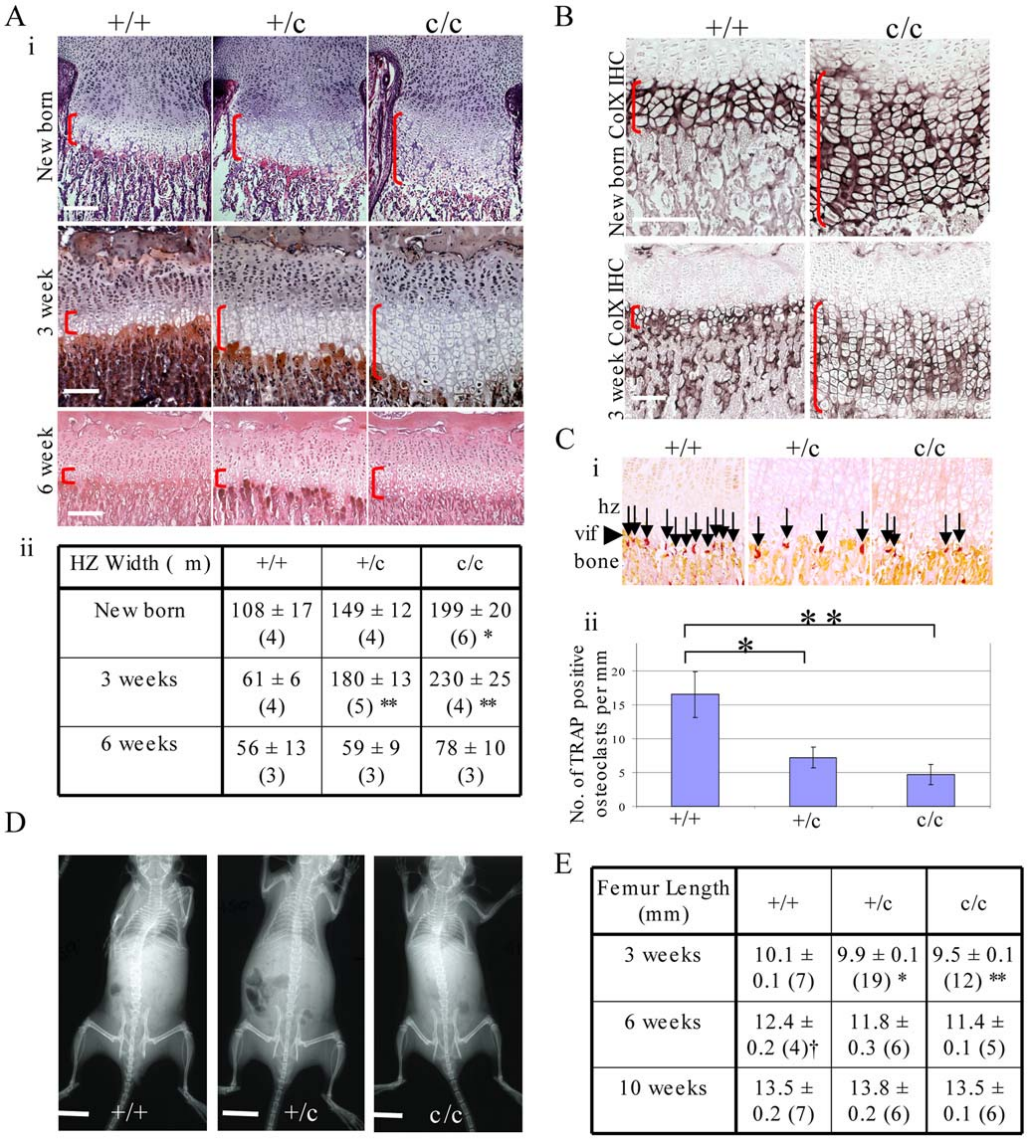
Characterisation of the *ColXTg^cog^* mouse phenotype. (A) Characterisation of the tibial hypertrophic zone expansion in new born, 3 week and 6 week old mice: (i) H & E staining at the different time points (vertical red brackets indicates the extent of the hypertrophic zone, horizontal white scale bar = 100 µm); (ii) Measurement of hypertrophic zone widths in the different genotypes (mean±SEM (n), *p<0.05 for c/c vs +/+, **p<0.001 for pooled c/c and +/c vs +/+. (B) Collagen X immunohistochemistry in new born and 3 week old mice (vertical red brackets indicates the extent of the hypertrophic zone, horizontal white scale bar = 100 µm). (C) TRAP staining for osteoclasts at the vascular invasion front (vif - arrow heads, hz = hypertrophic zone): (i) Vertical arrows indicate positively stained cells (red/brown colour); (ii) Histogram depicting number of positive cells per 1 mm of vascular invasion front. *p<0.05, **p<0.01 (n = 3 for each genotype). (D) X-ray images of 6 week old mice, scale bar = 1 cm. (E) Femoral length measurements (mean±SEM (n): *p<0.05 vs c/c; **p<0.01 vs +/+; ^†^p<0.05 vs pooled c/+ and c/c).

New born and 3 week old +/c and c/c mice exhibited an expanded hypertrophic zone (Figure 9A). The hypertrophic zone in new born c/c mice was 80% thicker than +/+ controls (p<0.05). The +/c animals exhibited intermediate growth plate thicknesses. At 3 weeks of age, the hypertrophic zones of +/c and c/c animals were increased 3- and nearly 4-fold respectively compared to controls (Figure 9A; p<0.001 vs +/+ for each group). However, at 6 weeks of age, the hypertrophic zone expansion apparent at younger ages in the +/c and c/c mice had largely resolved (Figure 9A).

Expression of Tg^cog^ protein in hypertrophic chondrocytes did not affect the normal secretion of collagen X (Figure 9B). TRAP staining revealed significant reductions in the numbers of osteoclasts localised to the vascular invasion front in mice expressing the Tg^cog^ protein (Figure 9C). X-ray analysis (Figure 9D) revealed that at 3 weeks, the femurs of c/c mice were significantly shorter than +/+ controls and at 6 weeks of age, the femurs of +/c and c/c mice were approximately 6% shorter than the +/+ controls (p<0.05; Figure 9E). However, by 10 weeks of age, long bone growth in *ColXTg^cog^* mice was indistinguishable from that of the age-matched controls (Figure 9E). A slight distortion of the pelvis was apparent in +/c and c/c mice (Figure 9D).

### The disruption of gene expression induced by Tg^cog^ is similar to that caused by mutant collagen X

**Figure 10:**
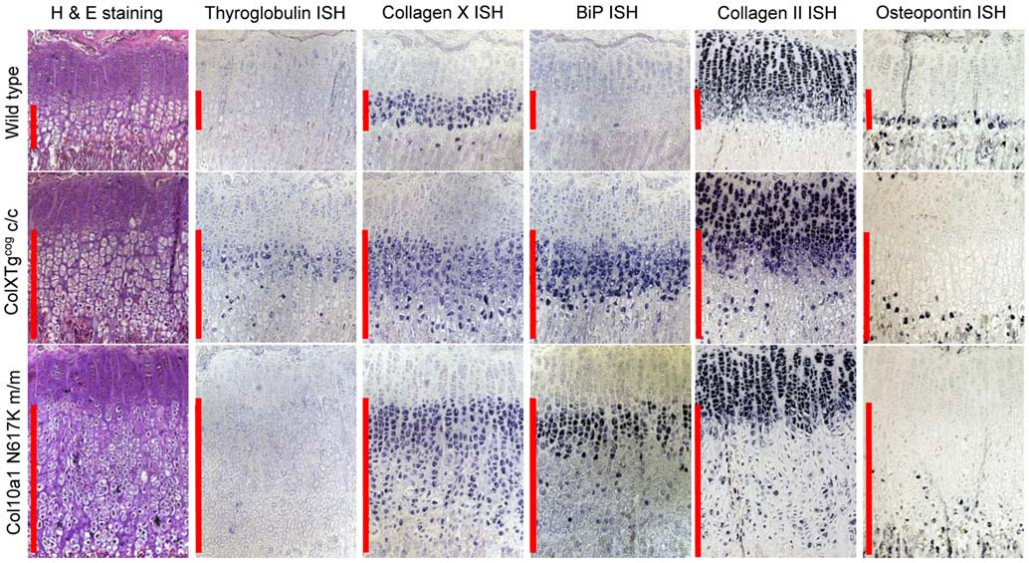
*In situ* hybridisation comparing the *ColXTg^cog^* and MCDS phenotype. Tibial growth plates from 3 week old mice homozygous for either the *ColXTg^cog^* or *Col10a1* p.Asn617Lys mutations were analysed for the expression of mRNAs encoding thyroglobulin, collagen X, BiP, collagen II, and osteopontin as indicated. The vertical red bars delineate the hypertrophic zones.

Direct comparison of the growth plates of 3 week old c/c or m/m mice revealed that the expansion of the hypertrophic zone appears greater in the m/m line (Figure 10). Thyroglobulin expression was only apparent in the hypertrophic zone of the c/c mouse and was not detectable in either +/+ or m/m mice (Figure 10). Thyroglobulin expression (which is driven by the *Col10a1* promoter in the growth plate of the c/c mouse) was induced at the boundary between the proliferative and hypertrophic zones. The expression of thyroglobulin was subsequently down-regulated and then up-regulated in an uncoordinated fashion further down the expanded hypertrophic zone in the c/c mouse growth plate (Figure 10). Similarly, the wildtype collagen X expressed by the c/c mouse was induced at the boundary of the proliferative and hypertrophic zone but then down-regulated and subsequently up-regulated in sporadic cells in the lower part of the expanded hypertrophic zone. The disruption of collagen X expression appeared greatest in the m/m mouse growth plate. BiP expression, was markedly up-regulated in the upper hypertrophic zone of the c/c mouse in a similar fashion to that of the m/m mouse (Figure 10). In each line, BiP expression appeared to mirror the expression of the mutant, ER-stress inducing protein (Tg^cog^ and collagen X respectively). Collagen II expression, which is normally down-regulated in the hypertrophic zone was subsequently re-expressed by cells distributed sporadically in the lower hypertrophic zone of the c/c mouse in a similar but less severe fashion to that seen in the m/m mouse (Figure 10). The normal expression of osteopontin by only the most terminal of the hypertrophic chondrocytes was disrupted in the c/c growth plate in a similar fashion to the m/m mouse. Sporadic cells scattered throughout the lower half of the expanded hypertrophic zone turned on osteopontin expression in an uncoordinated fashion (Figure 10).

## Discussion

MCDS is a genetically dominant metaphyseal chondrodysplasia that results in a relatively mild dwarfism. In spontaneously occurring forms of MCDS in man [13] and pig [28], an expansion of the collagen X-expressing hypertrophic zone of the growth plate is associated with the decreased rates of longitudinal bone growth. In wt/m mice, a decrease in bone length accompanied by a growth plate hypertrophic zone expansion was apparent at 3 weeks of age compared to controls (Table 1; Figure 3). This is close to the age that peak growth rates are achieved in mice [29]. A more severe pathology was apparent in m/m mice (Table 1; Figure 2). The pelvis distortion apparent in m/m mice (Figure 2) was present at birth and was not affected by increased rates of exercise stimulated by the provision of wheel running facilities (data not shown). The pelvis distortion appears to result from the decreased rates of growth from the complex organisation of multiple growth plates that form this bone and similar distortions are exhibited by mouse models of diastrophic dysplasia and pseudoachondroplasia [14],[30].

Immunohistochemical localisation of collagen X in the m/m mice revealed intracellular retention of the mutant collagen X in the upper hypertrophic zone followed by a delayed and limited secretion in the lower part of the zone (Figure 4). These findings are in agreement with cell culture analyses which demonstrated that the collagen X with the p.Asn617Lys and other MCDS missense mutations misfold in the ER, are poorly secreted and induce increased levels of ER stress with a resulting UPR [10]. An increased level of ER stress within the hypertrophic zone of mice expressing the mutant collagen X was apparent from the up-regulation of mRNA encoding BiP (Figure 4B), a key indicator of the UPR [31],[32]. Western blotting of mutant mouse growth plate extracts revealed further evidence of an UPR including elevated levels of BiP protein as well as induction and cleavage of ATF6. Paradoxically, BiP protein levels appeared slightly more elevated in the growth plate extracts from wt/m rather than m/m collagen X mutant mice (Figure 5) as was also apparent in the equivalent western blots from the *ColXTg^co^* ^g^ mouse (Figure 8). In the wt/m growth plate, most of the hypertrophic chondrocytes expressed increased levels of BiP, based on *in situ* hybridisation, whereas in the m/m animals, the majority of cells in the (lower) hypertrophic zone did not express increased BiP (Figure 4). Whole growth plates were used to prepare the protein extracts and a fixed quantity of protein (20 µg) loaded per track. 4). Since a greater proportion of the total cells in the wt/m compared to m/m growth plate were expressing high levels of BiP, the levels of BiP protein in the wt/m track (Figure 5) appear marginally higher. It is noteworthy that recent studies of mouse lines expressing randomly integrated MCDS-associated mutant collagen X transgenes (*Col10a1.13del* and p.P620fsX621) also reported an expanded hypertrophic zone exhibiting clear signs of increased ER stress [11],[13]. Not only do cells in the expanded hypertrophic zone of all of these MCDS mouse lines exhibit an UPR but gene expression patterns are disrupted in a similar fashion including fluctuations in steady-state mRNA levels for collagens X and II and osteopontin (Figure 4 and [11],[13]). It has been suggested that the disruption in gene expression patterns seen in the MCDS growth plate represents a reprogramming of hypertrophic chondrocyte differentiation where the chondrocyte adopts a pre-hypertrophic phenotype in order to decrease the expression of the mutant gene and thus reduce the level of ER stress whilst also slowing endochondral bone growth rates [11].

The expression of VEGF by hypertrophic chondrocytes is known to be a key factor for controlling the rate of vascular invasion of the growth plate and a suppression of VEGF signalling causes an expanded hypertrophic zone [6],[25]. We therefore hypothesised that if VEGF expression was affected as part of the generalised disruption to terminal hypertrophic chondrocyte differentiation described above and previously [11],[13], the resulting reduced rate of vascular invasion and accompanying osteoclast erosion of the mineralised growth plate [33] would account for the expansion of the hypertrophic zone. VEGF expression was reduced in the m/m compared to wt/wt hypertrophic zone resulting in a significant reduction in the recruitment of osteoclasts to the vascular invasion front (Figure 6). The hypertrophic zone of the m/m mouse therefore expands due to the imbalance between the rate that cells proliferate, which is unaffected by genotype (see BrdU labelling, Figure 3C), and the VEGF-mediated rate at which the hypertrophic zone is degraded by osteoclasts at the vascular invasion front.

It has been generally accepted that diseases caused by mutations in genes encoding ECM proteins mainly result from either a matrix that is deficient in the protein (eg haploinsufficiency) or a defective ECM due to the presence of the secreted mutant product. The MCDS phenotype in the *Col10a1* mutant mouse is not due to a deficiency of wildtype collagen X within the ECM since mice which are either haploinsufficient or completely lacking the protein do not exhibit the characteristic expanded hypertrophic zone accompanied by reduced bone growth [13],[34],[35]. Other explanations, apart from ER stress, for how mutant collagen X could be causing MCDS include the possibility that collagen X interacts in the ER with another secreted component(s), the absence of which causes the phenotype. The intracellular retention of the mutant collagen X would lead to the retention of this second component in a similar fashion to that proposed for the role of mutant COMP in the pathogenesis of pseudoachondroplasia [8]. Mutations in this second locus might also be expected to cause an MCDS-like phenotype and such a second locus has been proposed (see [4]). Alternatively, the limited secretion of mutant collagen X (Figure 4A; m/m mouse) may create a defective extracellular environment that contributes to the phenotype.

It has been appreciated for some time that increased ER stress and the resulting UPR can play an important role in diseases involving tissues dedicated to the synthesis of extracellular proteins such as the pancreas or liver [e.g. 36]–[38]. Only recently, however, has evidence for the potential contribution of ER stress to disease pathogenesis across a range of connective tissue diseases begun to be appreciated. Several studies on mouse models of ECM diseases caused by mutations in secreted ECM proteins such as aggrecan [11], collagen I [16], collagen II [11], collagen IV [12], collagen X [11],[13], COMP [14], matrilin 3 [15] and mucin [17] have reported increased levels of ER stress and speculated upon the possible role played by ER stress in the respective disease mechanisms. In order to directly test the putative role of elevated ER stress and the UPR in the disease mechanism of the mutant collagen X mouse line described above, we generated a transgenic mouse in which an UPR was specifically targeted to hypertrophic chondrocytes. The Tg^cog^ protein used to induce the UPR had previously been shown to trigger a similar ER stress response to that described for collagen X mutations (Figure 5 and [10],[11]), and is efficiently retained intracellularly not only in thyrocytes but also when expressed in heterologous cell lines [26],[27].

Expression of the Tg^cog^ protein in hypertrophic chondrocytes induced an UPR very similar to that provoked by the mutant collagen X (cf Figure 5 and Figure 8). It is noteworthy that expression of Tg^cog^ did not cause any generalised secretion defect since the wildtype collagen X synthesised in the same cells appeared to be secreted normally with no evidence of intracellular retention (Figure 9B). Nevertheless, the Tg^cog^ -expressing mouse exhibited an expanded growth plate hypertrophic zone with decreased numbers of osteoclasts at the vascular invasion front and decreased rates of bone growth (Figure 9), a phenotype reminiscent of the mutant collagen X mouse (Figure 2 and Figure 3). In addition, Tg^cog^ caused changes in the expression patterns of other genes in the hypertrophic zone similar to those produced by MCDS-causing mutant forms of collagen X reported here and elsewhere (Figure 10 and [11],[13]). These data from the *ColXTg^cog^* mouse demonstrate that the predominant pathological features of MCDS can be phenocopied by simply elevating ER stress and provoking an UPR in hypertrophic chondrocytes. We therefore conclude that potential extracellular consequences of the secretion of mutant collagen X do not appear to play a major role in the pathogenic mechanism. Furthermore, since the wildtype collagen X synthesised by the *ColXTg^cog^* mouse was not retained but secreted normally (Figure 9B), the defective secretion of a putative binding partner for collagen X seems unlikely as a potential pathogenic factor. The phenotype of the *ColXTg^cog^* mouse demonstrates mechanistically the central role played by ER stress in the MCDS disease process.

How might the UPR be inducing the disruption in hypertrophic chondrocyte differentiation resulting in the MCDS phenotype? In both the *Col10a1* p.Asn617Lys and *ColXTg^co^* ^g^ mouse lines, hypertrophic differentiation appeared to start normally with the up-regulation of collagen X expression (Figure 4B and Figure 10). However, in these mouse lines, the expression of the collagen X gene equates to the induction of increased ER stress and an UPR due to the synthesis of a mutant protein (collagen X and Tg^cog^ in the respective mouse models). Expression of either of the mutant proteins correlated directly with the induction of mRNA for BiP (Figure 10) which is indicative of an UPR. Subsequently, the levels of mRNAs encoding the mutant proteins decreased dramatically (Figure 10) and this is presumably accompanied by a decreased rate of synthesis of the ER stress-inducing protein. The closely related reduction in BiP mRNA suggests that at this point, hypertrophic chondrocytes had established an UPR which was effectively dealing with the accumulated unfolded protein thus restoring homeostasis to the ER. Supporting this contention is the observation that a reduction in the intracellular accumulation of mutant collagen X and its delayed secretion (Figure 4A inset) appeared to correlate with the generalised down-regulation of BiP mRNA (Figure 4B). One possible explanation for how an UPR could cause a reduction in steady state levels of mRNA for particular genes may reside in the accompanying attenuation of general protein synthesis due to eIF2α phosphorylation [19]–[22] which could impact upon steady state levels of transcription factors driving the chondrocyte differentiation process. Decreases in these transcription factors would result in transcriptional attenuation for dependant genes. Such an explanation would account for why the wildtype collagen X gene expressed in the *ColXTg^cog^* mouse was down-regulated in a similar fashion to the Tg^cog^ transgene (Figure 10) and demonstrates that the effects of an UPR on transcription are generic and not in some way targeted at loci producing the mutant transcripts. The loss of these transcription factors would also explain the failure to induce significant expression of VEGF which, as discussed above, results in the expanded hypertrophic zone. Alternatively, the rapid degradation of a subset of ER-localised mRNAs catalysed by IRE1 in response to increased ER stress has been reported [39] and a similar phenomenon may be occurring in hypertrophic chondrocytes. The relationship between UPR and transcription is complex [40] and future work will address this relationship in these mouse lines.

Once the initial ER stress induced by the expression of mutant collagen X or the Tg^cog^ transgene has been dealt with, the chondrocytes appear to revert to a prehypertrophic phenotype characterised by the re-expression of collagen II (Figure 10), PTHrP receptor and Indian hedgehog ([11],[13] and data not shown) prior to re-initiating terminal differentiation including collagen X expression. This re-engaging of the differentiation process is not synchronised in the lower half of the expanded hypertrophic zones and gives rise to the characteristic ‘speckled’ pattern of gene expression seen. Completion of the terminal differentiation step, hypertrophy, is achieved in a highly synchronised fashion in the normal growth plate as evidenced by the expression of markers such as osteopontin by a one or two cell deep layer adjacent to the vascular invasion front (wildtype, Figure 10). However, individual cells scattered throughout the lower half of the expanded hypertrophic zones express osteopontin in a non-coordinated fashion (Figure 10 and [11],[13]). The disruption and delay in the terminal differentiation of hypertrophic chondrocytes including the reduced VEGF signalling (Figure 6 and Figure 10) caused by the UPR results finally in reduced rates of bone growth. It is noteworthy that for mice heterozygote for the collagen X mutation and mice hemi- or homozygous for the *ColXTg^co^* ^g^ allele, reduced rates of bone growth could only be detected in juvenile, rapidly growing animals (Table 1 and Figure 9 respectively). In all these genotypes, the bone lengths achieved by 10 weeks of age were not significantly different from wildtype controls meaning that the decreased rates of bone growth exhibited in juvenile mice were compensated for as bone growth rates decreased into adulthood (Table 1). In the case of the *ColXTg^co^* ^g^ mouse line, it is possible that the regression of the pathology is in part due to transgene silencing in maturing animals as reported for the *Col10al.13 del* mouse [11]. However, Tg^cog^ and elevated BiP expression were still detectable in animals at 6 weeks of age (data not shown). In addition, the promoter used in the current study to drive Tg^cog^ expression was significantly longer than that used in the previous study [11] and contained all the sequences necessary to direct *bona fide* collagen X expression [41]. The regression of pathology in the wt/m *Col10a1* mutant mouse is unlikely to be due to gene silencing since in this line, expression was driven by the endogenous collagen X promoter. It seems more likely that, as discussed below, regression of pathology is related to reduced levels of ER stress being experienced in the growth plate as the rates of growth decrease into adulthood. In humans it has also been reported that some of the milder skeletal changes associated with MCDS appear to regress with age [42].

These observations raise several important points and questions. Firstly, how does disease severity relate to the level of ER stress experienced? Several different factors may influence the level of ER stress experienced by a cell. Previous studies have clearly demonstrated a gene dosage effect relating the level of mutant collagen X expression, and therefore the level of ER stress experienced, to the disease severity [11],[13]. Likewise, in this study, wt/m and +/c mice had significantly milder phenotypes than their homozygote counterparts. Natural, growth-related alterations in the level of gene expression may account in part for the transient expansion of the hypertrophic zone apparent in growth plates of wt/m and +/c animals at 3 weeks of age as peak growth rates are achieved (Figure 3 and Figure 9). In addition, the milder phenotype of mice homozygous for the *Tg^cog^* allele which exhibit no permanent dwarfing in comparison with the mutant *Col10a1* allele may relate to the fact that the former transgene is expressed at only around one third the level of the latter. It is also possible that different mixed genetic backgrounds account for some of the variations in phenotype noted between lines such as the earlier onset of hypertrophic expansion apparent in the +/c mice at birth. Other factors that will influence the level of ER stress experienced by a cell include: the nature of the mutation which may interfere with protein folding in different ways producing misfolded proteins that are more or less easily dealt with by the cell; and the behaviour of the misfolded protein is also critical since, as recently demonstrated for mutant forms of type I collagen, aggregates in the ER trigger autophagy whereas non-aggregated forms are subject to ER associated degradation [43]. In this manner, different mutations in the same protein may produce different levels of ER stress and therefore account, at least in part, for the spectrum of clinical phenotype seen in MCDS [4].

Secondly, the transient nature of the phenotype in our heterozygote mouse lines coupled to the fact that some of the skeletal pathology associated with MCDS in humans appears to regress with age raises the possibility that relatively modest decreases in the level of ER stress may produce significant improvements in clinical severity. Methods of reducing ER stress using chemical chaperones [44] or drugs designed to reduce ER stress by targeting pathways that control the stress response itself or mechanisms of degradation for misfolded proteins [45] are being developed and these represent possible novel therapeutic opportunities for treating MCDS. Finally, the fact that increased ER stress and the resulting UPR is of pathogenic significance in chondrocytes *in vivo* (as shown here and [11],[13]) supports the contention that such stress may play a significant role in the pathogenesis of many of the connective tissue diseases that are caused by mutations in ECM or related genes [11]–[18].

## Materials and Methods

### Generation of mutant mice

*Col10a1* p.Asn617Lys mouse line: Gene targeting was performed as described [46]. Briefly, an SV129 DNA library (RPCI) was screened and the PAC clone number 628-I3 identified as encoding the *Col10a1* gene. The BglII-BamHI 470 bp fragment of DNA flanking the Asn617 position (nucleotide 1849–1851 of the cDNA sequence from the ATG start codon) was amplified using primers: Forward: CAA GAT CTG GTA TCT TTA CCT GTA AGA TCC and Reverse: GAG GAT CCT CAC ATA CCC ACT GTT ACT G, and subcloned into pGEM-Teasy as per manufacturers instructions. The single base pair change required to produce the p.Asn617Lys mutation (C1851A) was introduced using the QuikChange XL site directed mutagenesis kit (Stratagene) with the following primers (mutated base underlined): F: CGT TTG GGT AGG CCT GTA TAA GAA AGG CAC GCC TAC GAT GTA CAC and R: GTG TAC ATC GTA GGC GTG CCT TTC TTA TAC AGG CCT ACC CAA ACG. The presence of the mutation was confirmed by direct sequencing. The 5′ 6194 bp BglII fragment was ligated upstream of the mutated fragment and the 2045 bp BamHI/EcoR1 fragment encoding downstream sequence was inserted 3′ to it (see Figure 1A). A *LoxP*-flanked Neo TK selection cassette was ligated into the unique Sgf1 site located approximately 200 bp 3′ to the stop codon. The construct was then linearised by digestion with NotI, electroporated into mouse R1 (SV129) ES cells and 360 G418 resistant clones were picked and frozen down as described [46]. Homologously recombined clones were screened for by EcoRV digestion and Southern blot analysis with the external probe indicated (Figure 1A and 1B). Homologously recombined clones were subsequently transiently transfected with the *Cre* recombinase gene and selected with FIAU to remove the floxed Neo TK selection cassette as described [15]. Correctly targeted clones were used to generate germline chimeras [46]. Mice carrying the *Col10a1* mutation (which was confirmed by direct sequencing, Figure 1D) were maintained on an SV129/C57Bl6 mixed background. Genotyping was performed using primers (F: GAT TTA TGG TGA GTT AGA GTC and R: GTG AGC ACT TCC TGT CAA GC) flanking the *LoxP* site (Figure 1A and 1C).

*ColXTg^cog^* mice: pBS was digested with KpnI and SacI to remove the multiple cloning site which was replaced with a double stranded oligo encoding the following sites: SgfI, XhoI, PmeI, NotI, SgfI. An 8276 bp XhoI fragment from PAC 628-I3 equivalent to base pairs 26809230 to 26817511 of mus musculus chromosome 10 genomic contig NT_039492, and including exons 1 and 2 and the 5′ portion of exon 3 was isolated. This region encompasses sequence 4.6 kb upstream of the start site and includes all the putative cis acting promoter and enhancer elements reported to drive specific hypertrophic chondrocyte expression [41]. The collagen X translation start site and two subsequent ATG's were removed by overlapping PCR. The 8.5 kb cDNA encoding the cog mutant form of thyroglobulin, including the start codon and polyA signal was subcloned in 2 stages. Firstly the 5′ 8 kb Pme1-Not fragment was subcloned followed by the 400 bp 3′ Not1 fragment containing the polyA addition signal. The construct was verified by sequencing across ligation boundaries, removed from the vector by digestion with SgfI, and the purified DNA used for pronuclear injection into donor blastocysts which were implanted into pseudopregnant foster mothers. Offspring were assessed for the presence of the transgene by PCR between the collagen X promoter and the Tg^cog^, as indicated (Figure 7A), with the following primers: ColX-GenF: GGA CTG TTG TGT GAG TGG and Tg^cog^-GenR: TTC CAT CTT CAG AGC ACT GG. Hemizygous (+/c) and homozygous (c/c) genotypes were determined by quantitating the relative levels of the transgene using the primers described above in real-time PCR (see below) on genomic DNA.

### Real-time PCR

Expression levels of Tg^cog^ mRNA in transgenic mice were assessed as follows: New born rib growth plates were placed in RNAlater (Sigma), homogenised, put through a QIAshredder (Qiagen), and RNA extracted using the Qiagen RNAeasy kit (Qiagen). The RNA was DNaseI (Ambion Inc.) treated and Superscript III reverse transcriptase with random hexamers (Invitrogen Ltd.) was used to generate cDNA. Real-time analysis of the relative expression of Tg^cog^ to that of collagen X was performed using the following intra-exon primer pairs: Tg-RT-F: AGG CAT GTG CAG TGT GAT GG and Tg-RT-R: GGT ACT GTG CTA GCA CTG G, and ColX-RT-F: CTT CCT GTC AAG CTC ATC C and ColX-RT-R: TAG GAT TGC TGA GTG CTC C. Wildtype genomic DNA was used as one control together with a no-template control to check for contamination. All reactions were performed in duplicate using SYBR Green Kit on an ABIPrism 7000 sequence detector system (Applied Biosystems Ltd).

### Skeletal analyses

Skeletal preparations of newborn mice were prepared as described [47]. X-rays of mice were produced using a Flaxitron X ray specimen radiography system (Flaxitron) and X-ray film (Kodak). Bone length measurements were taken from radiographic images. All the measurements were analysed by ANOVA for statistical significance.

### Histology and immunohistochemistry

Bone samples were fixed overnight in either 95% ethanol/5% acetic acid or in ice-cold 4% PFA. They were then decalcified in 0.25 M EDTA, embedded in paraffin wax and sectioned sagittally and mounted on positively charged glass slides. For haemotoxylin and eosin (H & E) staining, the slides were dewaxed in xylene, rehydrated and H and E stained using a ThermoShandon Ltd automated stainer, dehydrated in an ethanol gradient, cleared in xylene and mounted using a xylene-based mounting solution.

Hypertrophic zone width measurements were taken in the central part of the image. The start of the zone was defined as the point at which the disc shape cells of the proliferative zone started to round up and become larger, and the end of the zone taken as the vascular invasion front. Measurements were performed using the Photoshop Ruler Tool on images of known magnification. For each animal, three separate sections spaced at least 75 µm apart were averaged.

Immunohistochemistry against collagen X was carried out on ethanol/acetic acid-fixed samples. A rabbit polyclonal antibody to recombinant mouse collagen X NC1 domain was produced (Eurogentec). Briefly, a Antibodies to BrdU, and the myc epitope (Roche -) were used on PFA fixed tissue as described [48]. BrdU labelling was carried on 3 week old mice which were injected intraperitoneally with 100 mg BrdU (Sigma) per kg body weight, and sacrificed 2 hours later. Tibias were fixed in ice-cold 4% PFA, demineralised in 4% PFA containing 0.25 M EDTA and sectioned as described above. Antigen unmasking was performed in 4 M HCl for 15 minutes, neutralised with 0.1 M borate buffer. The number of BrdU-labelled cells was expressed as a proportion of the total population of cells in the proliferative zone which was defined as the region of the growth plate in which chondrocytes are disc shaped and form columns. Images were prepared using a Carl Zeiss Axiocam Colour CCD camera with associated Axiovision software and processed using Adobe Photoshop.

### *In situ* analysis

*In situ* hybridisation using ^35^S-labelled RNA probes was carried out as described [49]. DIG labelled colourimetric *in situ* was performed as described [50]. Tibias from 3 week old animals were fixed in ice-cold 4% PFA, demineralised in 4% PFA containing 0.25 M EDTA and sectioned as described above. The collagen II probe was a 600 bp insert encoding 3′ UTR from I.M.A.G.E cloneID 735113; the collagen X probe was a 900 bp fragment the 3′ end of the coding sequence; the osteopontin probe was the 1 kb fragment from I.M.A.G.E cloneID 1481264; and the BiP probe was a 350 bp fragment from I.M.A.G.E cloneID 6334883. The VEGF probe was as described [51]; the thyroglobulin probe was a 700 bp cDNA fragment amplified using primers F: TTG TAG ATC CAT CCA TCA AGC and R: GTG ACT ACG ATG AAG TTG C. cDNA probes were cloned into pT7T3, linearised and transcribed using the appropriate restriction enzyme and RNA polymerase respectively.

### Western blotting

Rib growth plate tissue was dissected from 3 week old animals, homogenised and boiled in SDS loading buffer containing DTT and centrifuged before loading (20 µg protein per sample) on SDS–PAGE gels. The protein concentration of extracts was assayed using the Pierce bicinchoninic acid (BCA) protein assay kit with a bovine serum albumin standard curve. The gel was electroblotted onto a nitrocellulose membrane, which was blocked overnight with 2% skimmed milk powder in PBS-T. Primary antibodies (Grp78/BiP - Santa Cruz (sc-1051) and ATF6 - Imgenex (IMG-273)) were diluted 1∶500 in the blocking solution and the appropriate secondary antibody (goat anti-rabbit (1∶1000) or goat anti-mouse (1∶10 000) - ThermoFisher Scientific Ltd) was diluted in PBS-T. An ECL detection kit (Perkin-Elmer Inc.) was used to develop the blots according to manufacturer's protocol.

### Tartrate-resistant Acid Phosphatase (TRAP) staining

Osteoclasts at the vascular invasion front were stained using the Acid Phosphatase kit from Sigma. The protocol was performed as per the manufacturer's instructions, on PFA fixed sections. For each genotype, 3 animals were assessed and for each animal, three separate sections spaced at least 50 µm apart were analysed.
